# Coordinate activities of BRD4 and CDK9 in the transcriptional elongation complex are required for TGF*β*-induced Nox4 expression and myofibroblast transdifferentiation

**DOI:** 10.1038/cddis.2016.434

**Published:** 2017-02-09

**Authors:** Talha Ijaz, Mohammad Jamaluddin, Yingxin Zhao, Yueqing Zhang, Jayson Jay, Celeste C Finnerty, David N Herndon, Ronald G Tilton, Allan R Brasier

**Affiliations:** 1 Department of Biochemistry and Molecuilar Biology, University of Texas Medical Branch, Galveston, TX, USA; 2 MD-PhD Program, University of Texas Medical Branch, Galveston, TX, USA; 3 Institute for Translational Sciences, University of Texas Medical Branch, Galveston, TX, USA; 4 Internal Medicine - Division of Endocrinology, University of Texas Medical Branch, Galveston, TX, USA; 5 Sealy Center for Molecular Medicine, University of Texas Medical Branch, Galveston, TX, USA; 6 Shriner's Hospital for Children, University of Texas Medical Branch, Galveston, TX, USA; 7 Department of Surgery, University of Texas Medical Branch, Galveston, TX, USA

## Abstract

Transdifferentiation of quiescent dermal fibroblasts to secretory myofibroblasts has a central role in wound healing and pathological scar formation. This myofibroblast transdifferentiation process involves TGF*β*-induced *de novo* synthesis of alpha smooth muscle cell actin (*α*SMA)+ fibers that enhance contractility as well as increased expression of extracellular matrix (ECM) proteins, including collagen and fibronectin. These processes are mediated upstream by the reactive oxygen species (ROS)-producing enzyme Nox4, whose induction by TGF*β* is incompletely understood. In this study, we demonstrate that Nox4 is involved in *α*SMA+ fiber formation and collagen production in primary human dermal fibroblasts (hDFs) using a small-molecule inhibitor and siRNA-mediated silencing. Furthermore, TGF*β*-induced signaling via Smad3 is required for myofibroblast transformation and Nox4 upregulation. Immunoprecipitation-selected reaction monitoring (IP-SRM) assays of the activated Smad3 complex suggest that it couples with the epigenetic reader and transcription co-activator bromodomain and extraterminal (BET) domain containing protein 4 (BRD4) to promote Nox4 transcription. In addition, cyclin-dependent kinase 9 (CDK9), a component of positive transcription elongation factor, binds to BRD4 after TGF*β* stimulation and is also required for RNA polymerase II phosphorylation and Nox4 transcription regulation. Surprisingly, BRD4 depletion decreases myofibroblast differentiation but does not affect collagen or fibronectin expression in primary skin fibroblasts, whereas knockdown of CDK9 decreases all myofibroblast genes. We observe enhanced numbers and persistence of myofibroblast formation and TGF*β* signaling in hypertrophic scars. BRD4 inhibition reverses hypertrophic skin fibroblast transdifferentiation to myofibroblasts. Our data indicate that BRD4 and CDK9 have independent, coordinated roles in promoting the myofibroblast transition and suggest that inhibition of the Smad3-BRD4 pathway may be a useful strategy to limit hypertrophic scar formation after burn injury.

Cutaneous wound healing is a multistep process involving sequential phases of coagulation/inflammation, proliferation/re-epithelization and wound closure/remodeling.^1^ The proliferation phase is initiated by formation of granulation tissue providing a matrix for recruitment of activated myofibroblasts that have central role in matrix deposition, re-epithelialization and eventual wound closure.^2^ In certain injuries, notably burn injuries, an exaggerated response results in excessive extracellular matrix (ECM) deposition and hypertrophic scar (HTS) formation.^3^ HTS produces significant morbidity through pruritis, compression, anatomic deformity and decreased joint mobility for which few effective treatments are available.^4^ Consequently, there is a need to understand molecular mechanisms involved in HTS formation to modify the repair process and prevent these adverse outcomes.

Myofibroblasts, present in limited numbers in the normal skin, are found in overabundance in HTS after burn injury.^5^ Myofibroblasts express *α*SMA and actin-associated SM22*α*, providing contractile strength^6, 7^ as well as collagen (Col)-1 and -3 and fibronectin for ECM remodeling.^8^ TGF*β* is a potent cytokine upregulated after injury that promotes myofibroblast transdifferentiation.^9^ TGF*β*1 binds to the TGF*β*RII receptor (T*β*RII) which heterodimerizes with T*β*RI/ALK5, a complex that recruits and phosphorylates transcription factors Smad2 and Smad3 on serine residues.^10, 11^ Phospho-Smad2/3/4 complexes translocate into the nucleus to bind Smad-binding elements (SBEs) in gene regulatory elements to promote myofibroblast programming.^12, 13, 14^

In pulmonary and cardiac fibroblasts, TGF*β* induces generation of reactive oxygen species (ROS) that mediate myofibroblast transdifferentiation.^15, 16^ TGF*β* upregulates NADPH oxidase (Nox) 4 that utilizes electrons from NADPH to generate superoxide before rapidly converting it into hydrogen peroxide.^17^ Unlike other members of Nox family of enzymes, Nox4 is regulated at the level of gene expression.^18^ Suppression of Nox4 decreases myofibroblast formation and fibrosis in lung, liver, kidney and cardiac injury models.^15, 19, 20, 21^ However, fibroblasts are phenotypically and functionally distinct in different organ types.^22^ In this study, we investigated whether dermal fibroblasts also utilize Nox4 to promote myofibroblast transdifferentiation program.

Myofibroblast transdifferentiation occurs within the context of an inflammatory response. Our group has demonstrated the central role of regulated transcription elongation in mediating inducible genes in inflammatory and growth factor pathways.^23, 24, 25^ In this process, signal transduction pathways activate a complex of cyclin-dependent kinase 9 (CDK9) and bromodomain-containing protein 4 (BRD4) to promote active transcriptional elongation.^26^ Here we report the finding that Nox4 is required for TGF*β*-induced transdifferentiation of human dermal fibroblasts (hDFs) into myofibroblasts via Smad3-dependent recruitment of BRD4 and CDK9. Both BRD4 and CDK9 were required for myofibroblast phenotype, whereas only CDK9 was required for ECM expression. Finally, we demonstrate that HTS fibroblasts have constitutive Smad3-BRD4 activation and inhibition of BRD4 reverses the myofibroblast phenotype. These data suggest a cooperative, independent roles of CDK9 and BRD4 in the HTS myofibroblast phenotype.

## Results

### TGF*β* promotes dermal fibroblast Nox4 expression and myofibroblast transdifferentiation

**Figure 1:**
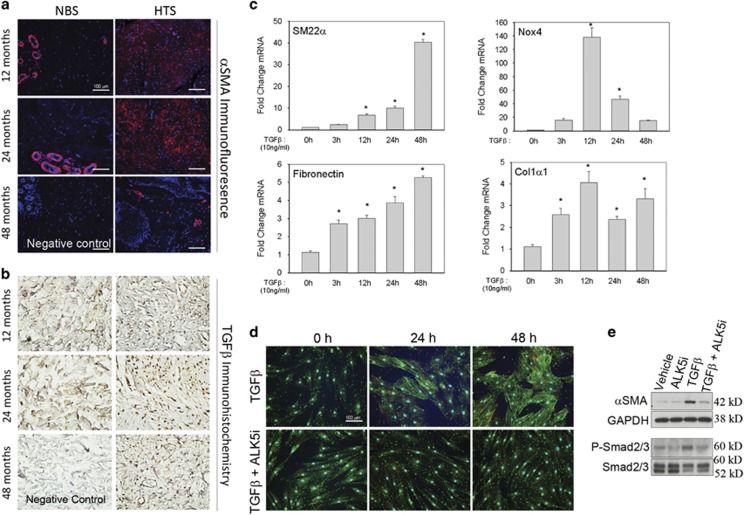
TGF*β* induces dermal fibroblast Nox4 expression and promotes transdifferentiation to myofibroblast. (**a**) Immunofluorescence detection of αSMA+ myofibroblasts in skin biopsies taken from burn patients, 12–48 months after injury, at the site of HTS formation and from the adjacent NBS. Positive staining for *α*SMA is in red and counterstaining for nuclei is in blue. Note that myofibroblasts have almost completely disappeared from the dermis 48 months after injury. There is some positive staining of glandular smooth muscle cells and vascular smooth muscle cells with anti-αSMA antibody in NBS and HTS sections, which serves as an internal positive control. Scale bar represents 100 microns. (**b**) IHC for TGF*β* in HTS and NBS tissue sections from burn patients. Positive staining for TGF*β* is brown and counterstaining for nuclei is blue. Most of the positive staining for TGF*β* was observed in HTS biopsies at 12–24 months in the same location as the highest amount of *α*SMA+ staining. Scale bar represents 100 microns. IHC was performed on up to three tissue sections from a patient at each time point. Representative images are shown. (**c**) hDFs were stimulated with TGF*β* (10 ng/ml) for 0–48 h, and changes in gene expression for SM22*α*, Nox4, fibronectin and Col1*α*1 were analyzed via quantitative real-time PCR. Gene expression was normalized to DNA polymerase *β* mRNA. Data are presented as mean±S.E.M. This experiment was repeated three times. *P<0.05 *versus* 0 h. (**d**) hDF cells were seeded on coverslips, pretreated with 10 *μ*M ALK5i LY2157299 before being stimulated with TGF*β* (10 ng/ml) for up to 48 h. Immunostaining for *α*SMA (green) and staining for filamentous actin using AlexaFluor-568-conjugated phalloidin (red) was performed. αSMA+ cells (green or yellow fibers) were considered to be myofibroblasts. Scale bar represents 100 microns. This experiment was repeated twice and similar results were observed. (**e**) hDF cells were pretreated with vehicle or 10 *μ*M ALK5i and then stimulated with TGF*β* (10 ng/ml). Whole-cell extracts were collected after 24 h and western blotting analysis was performed to analyze *de novo α*SMA production and phospho-Smad2/3. GAPDH and Smad2/3 served as loading controls. Experiment was repeated twice and similar results were obtained

To determine the extent of myofibroblast population in non-burn skin (NBS) and in HTS, we performed immunofluorescence staining for the myofibroblast marker *α*SMA^27^ on skin biopsies of patients taken 12–48 months following burn injury (Figure 1a). Few myofibroblasts were present in the NBS biopsies (Figure 1a). By contrast, myofibroblasts were highly abundant in the deep dermal layer of HTS at 12–24 months but disappear by 48 months after injury. We also observed robust TGF*β* staining in the deep dermis at 12–24 months (Figure 1b), suggesting that the myofibroblasts are maintained in a TGF*β*-rich microenvironment.

hDFs were stimulated with TGF*β* to determine the changes in Nox4 expression and the myofibroblast gene program. Sm22*α* increased steadily and peaked at 48 h (40-fold increase *versus* baseline), whereas Nox4 expression peaked earlier at 12 h (138-fold increase *versus* baseline), gradually declining thereafter (Figure 1c). Fibronectin and Col1*α*1 mRNAs also increased. To determine the role of canonical TGF*β* signaling, we examined the effects of a T*β*RI/ALK5 inhibitor (ALK5i) on TGF*β*-induced *α*SMA stress fiber formation and gene expression. TGF*β* induced stress fibers in ~30% of the cells 24 h after stimulation and ~50% by 48 h (Figure 1d), while ALK5i completely blocked stress fiber formation (Figure 1d), phospho-Smad2/3 induction and *de novo α*SMA synthesis (Figure 1e). These results suggest that TGF*β* induces myofibroblast transdifferentiation via ALK5-mediated phospho-Smad2/3 activation.

### Nox4 inhibition blocks myofibroblast differentiation with limited effect on myofibroblast genes

**Figure 2:**
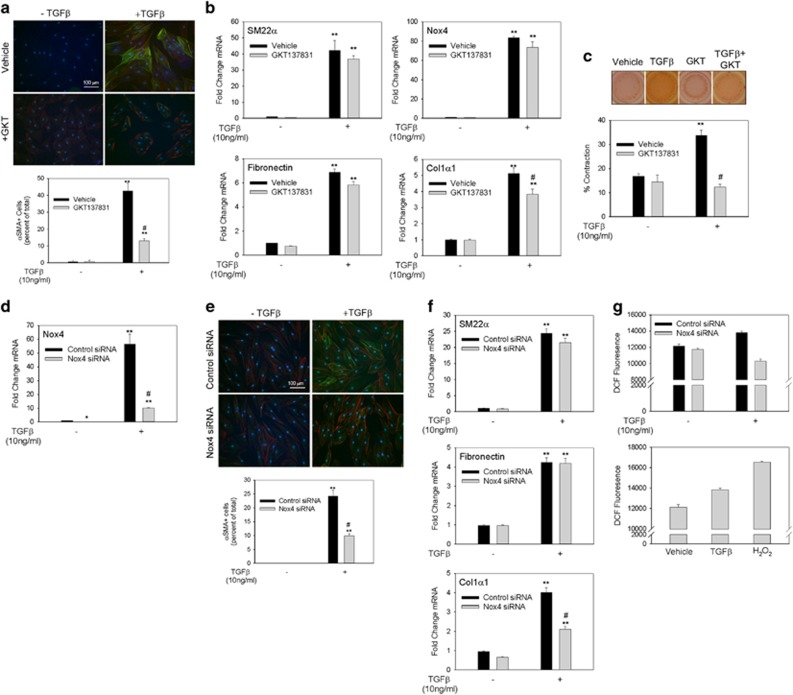
Nox4 inhibition with GKT137831 and Nox4 suppression with siRNA decrease dermal myofibroblast transformation. (**a**) hDFs were pretreated with vehicle or GKT137831 (20 *μ*M) for 1 h before stimulation with TGF*β* for 48 h. Immunofluoresence staining for *α*SMA (green) and filamentous actin stain with phalloidin (red) was performed. This experiment was repeated three times and the percentage of *α*SMA+ myofibroblasts were quantified over the three experiments. (**b**) hDF cells were pretreated with vehicle or GKT137831 (20 *μ*M) for 1 h followed by TGF*β* (10 ng/ml) for 24 h. Cellular mRNA was analyzed via quantitative real-time PCR (qRT-PCR) for changes in gene expression of SM22*α*, Nox4, fibronectin and Col1*α*1. (**c**) Collagen gel contraction assay was performed with hDF cells. Cells were treated with vehicle or GKT137831 (20 *μ*M) in the presence or absence of TGF*β* (10 ng/ml). Experimental groups were evaluated in triplicate. Change in gel surface area was determined after 48 h and is represented as the percentage of contraction of the gel. Experiments in panels (**a**−**c**) were repeated at least three times. Data are presented as mean±S.E.M. **P<0.05 *versus* −TGF*β*; ^#^*P*<0.05 *versus* vehicle+TGF*β* treatment. (**d**) After electroporation of control or Nox4 siRNAs, hDF cells were stimulated with TGF*β* (10 ng/ml) for 24 h. Total RNA was collected to analyze changes in gene expression by qRT-PCR. (**e**) hDF cells were electroporated with control or Nox4 siRNA and, after 3 days, were stimulated with TGF*β* for 48 h. Immunofluoresence staining for *α*SMA (green) and phalloidin staining for filamentous actin (red) was performed to detect myofibroblasts. The percentage of cells that transdifferentiate to myofibroblasts was determined. The average of three experiments is presented in the bar graph. (**f**) qRT-PCR for changes in SM22*α*, fibronectin and Col1*α*1 mRNAs. (**g**) To detect changes in ROS, DCF-DA assay was performed on cells electroporated with control or Nox4 siRNA and incubated with or without TGF*β* (10 ng/ml). As a positive control, hDF cells electroporated with control siRNA were treated with 4.8 nM H_2_0_2_. ROS detection assay was performed twice and each treatment group was evaluated in quadruplicates. Immunofluorescence staining in panel (**d**) and qRT-PCR experiments in panels (**e** and **f**) were performed three times each. All data are presented as mean±S.E.M. **P<0.05 *versus* −TGF*β*; ^#^*P*<0.05 *versus* control siRNA+TGF*β*

To probe the role of Nox4 in myofibroblast transdifferentiation, we used a potent small-molecule inhibitor, GKT137831.^28^ TGF*β* stimulation increased the fraction of *α*SMA+ myofibroblasts from 0.77 to 42.6% of total cells, which was reduced to 13.2% by GKT137831 pretreatment (Figure 2a). Surprisingly, increases in SM22α, Nox4 and fibronectin mRNAs were unaltered by GKT137831 treatment and only a slight reduction in Col1*α*1 was seen (Figure 2b). GKT137831 treatment prevented TGF*β*-induced collagen gel contraction over 48 h (Figure 2c).

To assess the specific role of Nox4, we depleted Nox4 mRNA by siRNA transfection. Nox4 expression was reduced by >80% in untreated hDFs and was significantly reduced in response to TGF*β* (Figure 2d). Nox4 silencing decreased TGF*β*-induced *α*SMA+ myofibroblast differentiation >50% *versus* control siRNA-treated cells (Figure 2e). Nox4 depletion did not affect TGF*β*-induced changes in myofibroblast genes SM22*α* or fibronectin but did inhibit the induction of Col1*α*1. To verify that Nox4 mediated ROS generation, we directly measured ROS by dichlorofluorescein (DCF) assay.^29, 30^ TGF*β* induced DCF fluorescence above baseline levels; this induction was completely blunted in Nox4-silenced cells without affecting basal levels (Figure 2f). This suggests that TGF*β*-induced ROS is mediated through Nox4.

### Smad3 regulates Nox4 and myofibroblast transformation

**Figure 3:**
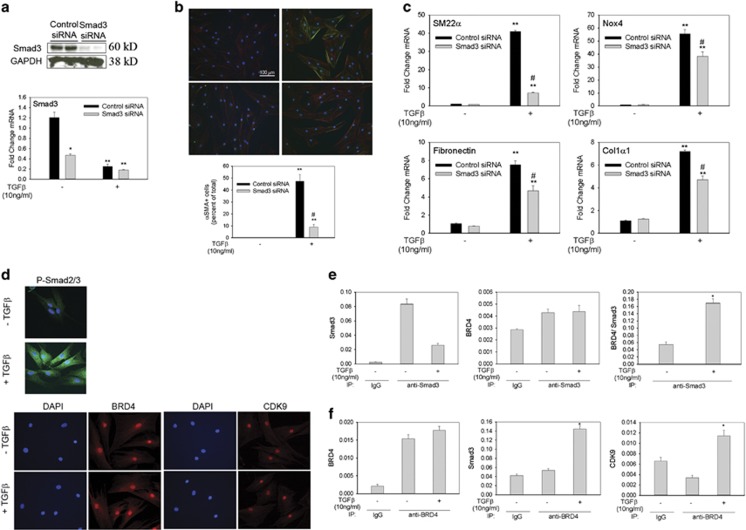
Smad3 regulates myofibroblast transdifferentiation and binds to BRD4 during TGF*β* stimulation. (**a**) hDFs were electroporated with control or Smad3 siRNA. After 72 h incubation, cells were stimulated with TGF*β* (10 ng/ml) for 48 h before analysis. Top panel, whole-cell extracts from hDF were analyzed for Smad3 content by western blotting. GAPDH was used as a loading control. Bottom panel, Smad3 mRNA expression was analyzed by quantitative real-time PCR (qRT-PCR). Immunofluoresence and qRT-PCR experiments were repeated at least three times. Western blotting was repeated twice. (**b**) hDF cells were fixed and immunostained for *α*SMA (green) and phalloidin for filamentous actin (red). Myofibroblasts were quantified in three separate experiments. Data presented are the mean of three experiments. (**c**) qRT-PCR of myofibroblast and ECM gene expression. Data in panels (**a**−**c**) are presented as mean±S.E.M. **P<0.05 *versus* −TGF*β*; ^#^*P*<0.05 *versus* vehicle+TGF*β* . (**d**) Immunofluoresence staining for Smad2/3, phospho-Smad2/3, BRD4 and CDK9 in hDF cells incubated with or without TGF*β* (10 ng/ml) for 24 h. DAPI was used to stain nuclei. Scale bar represents 100 microns. Immunofluoresence experiments were repeated twice and similar results were observed. (**e**) hDFs were treated with TGF*β* (10 ng/ml) for 24 h. Equal amount of cell lysates were immunoprecipitated with anti-Smad3 antibody and subjected to SID-SRM-MS analysis of Smad3 and BRD4 protein levels. Data are presented as the mean ratio of native to SIS peptides or as BRD4 signal normalized to Smad3. (**f**) hDF cells were treated with TGF*β* (10 ng/ml) for 24 h and cell lysates were immunoprecipitated with an anti-BRD4 antibody before being subjected to SID-SRM-MS analysis for BRD4, Smad3 and CDK9 proteins. Data are presented as the mean ratio of native to SIS peptide. All immunoprecipitation experiments were repeated three times. Bar graphs represent mean±S.E.M. *P<0.05 *versus* –TGF*β*

We next asked whether Smad3 was essential in Nox4 gene expression and in myofibroblast transdifferentiation. We observed a 70–80% depletion of Smad3 with siRNA (Figure 3a). Interestingly, we also noted that TGF*β* decreased Smad3 mRNA by >80% after 24 h, suggesting the presence of a negative feedback loop. Smad3 silencing decreased myofibroblast formation by >75% compared with control siRNA-treated cells (Figure 3b). In addition, Smad3 silencing blocked TGF*β* induction of SM22*α* mRNA, whereas the expression of Nox4, fibronectin and Col1*α*1 genes was reduced by 25–30% *versus* control siRNA-treated cells (Figure 3c).

### Smad3 directly interacts with the transcriptional co-activator BRD4

How TGF*β*-induced ALK5-Smad3 pathway activates myofibroblast programs is not known. Knowing that BRD4 and CDK9 have central roles in inducible inflammatory gene expression^23, 24, 25, 31, 32^ – a hallmark of the injury response^33^ – and reasoning that these co-activators may have a central role in the initial stages of wound healing, we asked whether Smad2/3 localizes in the same cellular compartments. Smad2/3 accumulated within the nucleus after TGF*β* stimulation as did phosphorylated Smad2/3 (Figure 3d). BRD4 and CDK9 were observed only in the nucleus, suggesting that their primary involvement may be in transcription regulation.

To determine whether Smad3 directly interacts with BRD4 and CDK9, whole-cell extracts were enriched for Smad3 by immunoprecipitation followed by quantitative measurement of BRD4 and CDK9 via selected reaction monitoring-mass spectrometry (SID-SRM-MS^34, 35, 36, 37^). We observed a significant enhancement of Smad3 in the Smad3 immunoprecipitates compared with control IgG, confirming target enrichment (Figure 3e, left panel). In a manner consistent with reduction of Smad3 mRNA expression in response to TGF*β* treatment (Figure 3a), the abundance of Smad3 protein in TGF*β*-treated immunoprecipitates was also reduced to ~25% of that of untreated cells (Figure 3e, left panel). SID-SRM-MS assays of the Smad3 immunoprecipitates showed that BRD4 associated with Smad3 in both unstimulated and stimulated cells (Figure 3e, middle panel). To account for the dramatic reduction in total Smad3 from TGF*β* treatment, we normalized BRD4 signal to Smad3 abundance in each immunoprecipitate. This analysis showed the normalized fraction of Smad3 bound to BRD4 increased by more than three-fold after TGF*β* treatment (Figure 3e, right panel). The reverse experiment, immunoprecipitating with anti-BRD4 antibody and quantifying bound Smad3, demonstrated a significant enrichment of BRD4 relative to control IgG (Figure 3f, left panel). In this case, the abundance of BRD4 was similar in control and TGF*β*-treated samples (Figure 3f, left panel), and we observed also that TGF*β* treatment increased Smad3 bound to BRD4 by ~3-fold (Figure 3f, middle panel) and increased CDK9 bound to BRD4 by ~4-fold (Figure 3f, right panel). These data suggest that TGF*β* increases the fraction of active Smad3 complexed with BRD4 and that BRD4 interacts with CDK9.

### Inhibition of BRD4 prevents myofibroblast transdifferentiation

**Figure 4:**
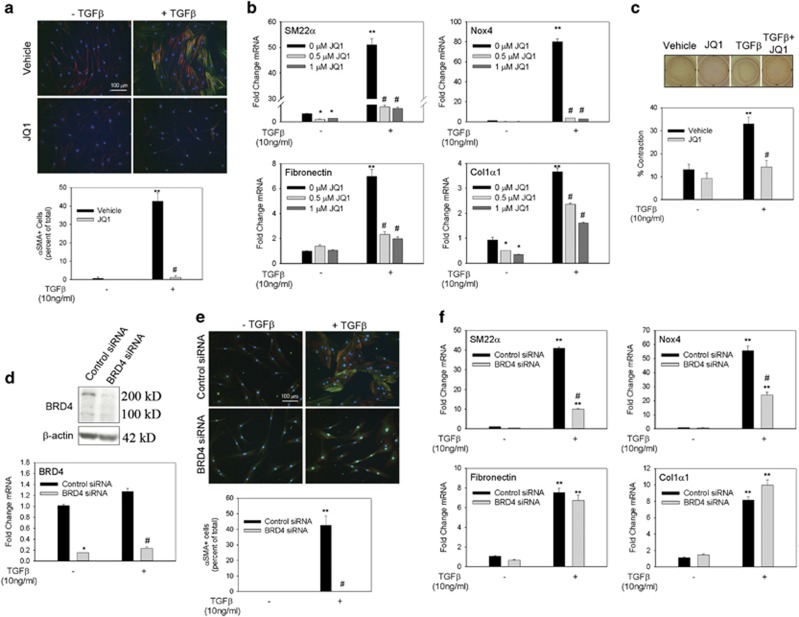
JQ1 treatment and BRD4 suppression with siRNA block myofibroblast transdifferentiation. (**a**) hDF cells were preincubated with 1 *μ*M JQ1 or vehicle for 1 h before stimulation with TGF*β* (10 ng/ml) for 48 h. Immunofluoresence staining for *α*SMA (green) and phalloidin staining for filamentous actin (red) was used to detect myofibroblasts. Myofibroblast cells (*α*SMA+) were quantified in three separate experiments. (**b**) hDF cells were preincubated with 0.5 or 1 *μ*M JQ1 for 1 h and then stimulated with TGF*β* (10 ng/ml) for 24 h. mRNA was analyzed via quantitative real-time PCR (qRT-PCR) to determine myofibroblast gene expression changes. (**c**) Collagen gel contraction assay was performed with hDF cells treated with vehicle or 1 *μ*M JQ1 in the presence or absence of TGF*β* (10 ng/ml). Gel surface area was measured at time 0 and 48 h and change in surface area is reported as the percentage of contraction of gel. The assay was performed in triplicate. Experiments in panels (**a**−**c**) were repeated at least three times. Data are reported as mean±S.E.M. *P<0.05 *versus* 0 *μ*M JQ1−TGF*β*; **P<0.05 *versus* −TGF*β* ^#^*P*<0.05 *versus* vehicle+TGF*β*. (**d**) hDF cells were electroporated with control or BRD4 siRNA. After 72 h, cells were incubated with or without TGF*β* (10 ng/ml) for another 48 h. Knockdown efficiency of BRD4 was determined via western blotting and qRT-PCR. *β*-Actin was used as a loading control. Western blotting analysis was repeated twice. (**e**) *α*SMA immunostaining (green) and phalloidin staining for filamentous actin (red) was performed to quantify myofibroblasts (aSMA+ cells). Myofibroblast cells were quantified in four separate experiments. (**f**) qRT-PCR analysis for myofibroblast genes. Data are presented as mean±S.E.M. *P<0.05 *versus* control siRNA−TGF*β*; **P<0.05 *versus* −TGF*β*; ^#^*P*<0.05 *versus* control siRNA+TGF*β*

To determine whether BRD4 was functionally required for Nox4 transcription and myofibroblast differentiation, we utilized JQ1, a competitive inhibitor of the BET bromodomain pocket used to bind to acetylated residues.^38^ JQ1 completely blocked the myofibroblast transition produced by TGF*β* treatment (Figure 4a). At concentrations as low as 0.5 *μ*M, JQ1 blocked not only TGF*β*-induced increases in SM22*α*, Nox4, fibronectin and Col1*α*1 mRNA but also decreased their constitutive expression. JQ1 also blocked TGF*β*-induced collagen gel contraction without significantly affecting baseline contraction (Figure 4c).

Using siRNA-mediated transfection, we achieved elimination of both the long and short BRD4 variants in western blotting and at least a 80% reduction in BRD4 mRNA expression (Figure 4d). BRD4 silencing prevented *α*SMA+ myofibroblast differentiation (Figure 4e) and diminished TGF*β* induction of SM22*α* and Nox4 by >50% (Figure 4f). Surprisingly, TGF*β*-induced transcription of fibronectin and Col1*α*1 was not affected by BRD4 knockdown, suggesting that another BET family member, in addition to BRD4, is involved in regulating these myofibroblast genes.

### Inhibition of CDK9 diminishes myofibroblast transdifferentiation

**Figure 5:**
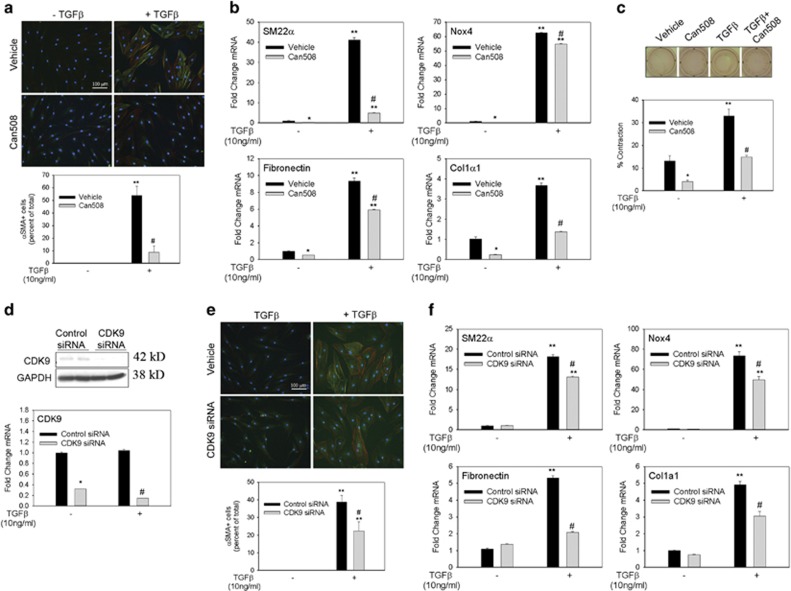
Inhibition of CDK9 with Can508 or CDK9 knockdown with siRNA decreases myofibroblast transformation. (**a**) hDFs were pretreated with Can508 (30 *μ*M) for 6 h before stimulation with TGF*β* (10 ng/ml) for 48 h. Immunofluorescence for *α*SMA (green) and phalloidin staining for f-actin (red) was performed. *α*SMA+ myofibroblast cells were quantified in three separate experiments. (**b**) Myofibroblast gene expression changes were analyzed via quantitative real-time PCR (qRT-PCR) in hDF cells pretreated with Can508 and stimulated with TGF*β* (10 ng/ml) for 24 h. (**c**) Collagen contraction assay was performed on hDF cells treated with vehicle or Can508 (30 *μ*M) in the presence or absence of TGF*β* (10 ng/ml). Change in surface area is reported as the percentage of contraction of the gels. Experiments in panels (**a**–**c**) were repeated at least three times. Data are represented as mean±S.E.M. *P<0.05 *versus* vehicle−TGF*β*; **P<0.05 *versus* −TGF*β*; ^#^*P*<0.05 *versus* vehicle+TGF*β*. (**d**) hDF cells were electroporated with control or CDK9 siRNA before being stimulated with TGF*β* (10 ng/ml) for 48 h. Top panel, whole-cell extracts extracts were assayed for CDK9 expression by western blotting. GAPDH was used as a loading control. Western blotting was repeated twice with similar results being observed. Bottom panel, qRT-PCR analysis of CDK9 mRNA. Data are presented as mean±S.E.M. of at least three experiments. (**e**) Cells were immunostained for *α*SMA and also stained for f-actin with phalloidin (red) to determine the myofibroblast population. Average of five experiments is represented in the bar graph. (**f**) mRNA was assayed for gene expression by qRT-PCR. Data are presented as mean±S.E.M. of at least three experiments. *P<0.05 *versus* control siRNA−TGF*β*; **P<0.05 *versus* −TGF*β*; ^#^*P*<0.05 *versus* control siRNA+TGF*β*

Because BRD4 complexes with CDK9, we next examined the role of CDK9 in myofibroblast differentiation using a CDK9-specific, small molecule inhibitor – Can508.^39^ Can508 successfully reduced TGF*β*-mediated *α*SMA+ myofibroblast transformation by 80% (Figure 5a). However, Can508 inhibited baseline Nox4 mRNA transcription by >90% but had minimal effect on Nox4 mRNA in response to TGF*β* stimulation (~15% reduction *versus* vehicle-treated cells, Figure 5b). In contrast, Can508 was a very potent inhibitor of SM22*α*, fibronectin and Col1*α*1 mRNA both at baseline and in response to TGF*β* stimulation, reducing gene expression by 40–90% *versus* vehicle-treated cells (Figure 5b). Further, Can508 treatment reduced contraction under basal conditions and during TGF*β* stimulation. These data suggest that CDK9 activity is functionally required for stress fiber formation, contractility and ECM synthesis.

As Can508 may have off-target effects, CDK9 was depleted in hDF cells to verify its role in myofibroblast differentiation. Transfection with CDK9 siRNA decreased CDK9 protein levels by >80% compared with control siRNA-transfected cells (Figure 5d); CDK9 mRNA expression was suppressed to a similar extent (Figure 5d). We found that CDK9 depletion inhibited TGF*β*-induced *α*SMA+ myofibroblast transformation by ~50% (Figure 5e). Furthermore, CDK9 knockdown diminished TGF*β* induction of Nox4, SM22*α*, fibronectin and Col1*α*1 by ~25–90% *versus* levels observed in control cells (Figure 5f). This demonstrated to us that CDK9 has a vital role in the transdifferentiation process by regulating myofibroblast gene transcription.

### TGF*β*-induced recruitment of p-Smad3 and CDK9 to the Nox4 promoter is dependent on BRD4

**Figure 6:**
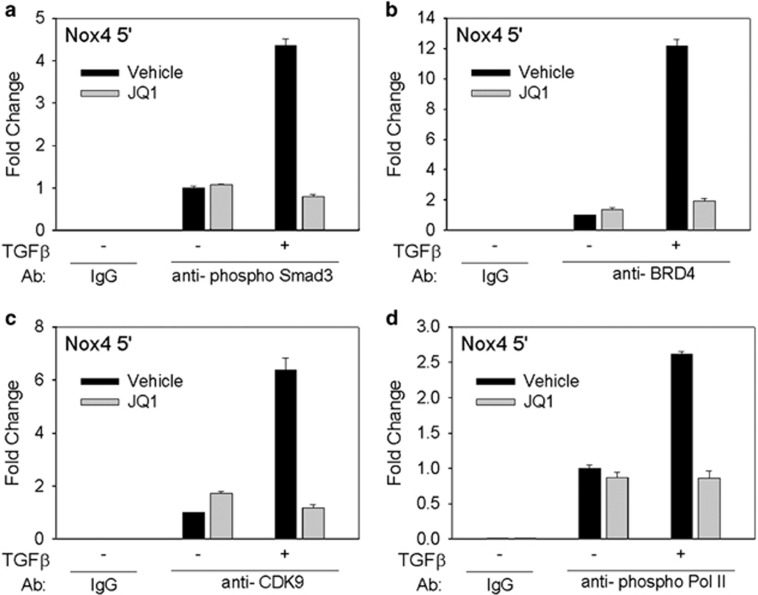
Increased accumulation of Smad3, BRD4 and CDK9 on Nox4 promoter after TGF*β* stimulation. hDFs were preincubated with vehicle or JQ1 for 1 h before being stimulated with TGF*β* (10 ng/ml) for 24 h. Cells were then subjected to XChIP analysis. (**a**) pSmad3 binding on the Nox4 promoter. Chromatin was immunoprecipitated with an antibody to p-Smad3; non-specific IgG was used as a negative control. Fold change was determined compared with −TGF*β* sample. (**b**) BRD4 binding. Experiment as in panel (**a**). (**c**) CK9 binding. (**d**) phospho-Ser2 RNA Pol II. For experiments in panels (**a**–**d**), data are presented as mean±S.E.M. from two independent experiments; similar results were obtained in each

As TGF*β*-induced transcription of Nox4 requires the transcription regulatory proteins Smad3, BRD4 and CDK9, we inferred that they must be recruited to the Nox4 proximal promoter. We examined the binding of these proteins to the Nox4 gene and their dependence on BRD4 acetylated histone reader using a two-step crosslink chromatin immunoprecipitation (XChIP) assay.^40^ TGF*β* treatment led to a 4.3-fold enrichment of activated p-Smad3 on Nox4 promoter (Figure 6a). Interestingly, inducible Smad3 recruitment was blocked by JQ1. Concordantly, TGF*β* induced a striking 12-fold increase in BRD4 binding (Figure 6b). JQ1 reduced BRD4 accumulation to 1.9-fold, indicating that BRD4 bromodomain interactions mediated its recruitment. TGF*β* also induced a 6.4-fold enrichment of CDK9 binding to Nox4 relative to untreated control hDFs, an effect that was also blocked by JQ1 (Figure 6c). Because BRD4 and CDK9 are both capable of phosphorylating Pol II at Ser2 to promote transcription elongation, we assessed the effect of TGF*β* on p-Ser2-Pol II enrichment. TGF*β* induced a 2.6-fold enrichment of p-Ser2-Pol II on the Nox4 gene; JQ1 treatment completely blocked this enrichment (Figure 6d). These data indicate that TGF*β*-induced recruitment of Smad3–BRD4–CDK9 complex is BRD4 dependent and that this complex is a key regulatory step in myofibroblast transdifferentiation promoting p-Ser2-Pol II formation and transcription elongation of Nox4 mRNA expression.

### Increased sensitivity of HTS fibroblasts to TGF*β* is mediated by BRD4

**Figure 7:**
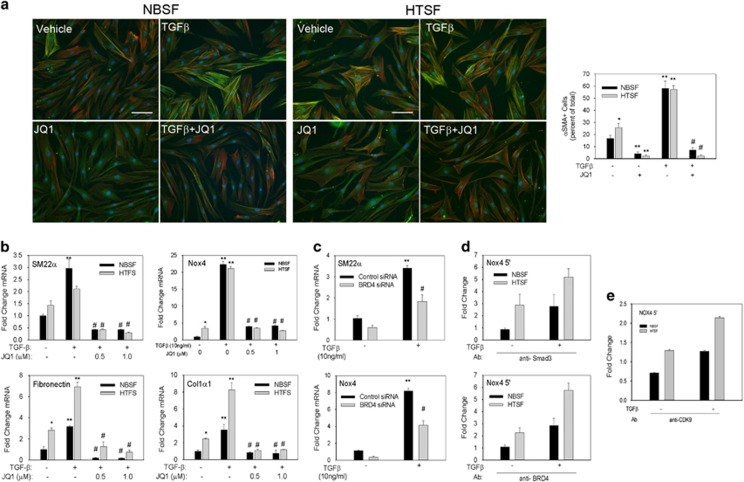
HTS fibroblasts have increased propensity for myofibroblast transformation, which can be blocked with JQ1. (**a**) HTS and NBS fibroblasts were preincubated with vehicle or JQ1 (1 *μ*M) for 1 h before being stimulated with TGF*β* (10 ng/ml) for 48 h. Cells were then immunostained for *α*SMA (green) and co-stained for f-actin with phalloidin (red). *α*SMA+ myofiboblasts were quantified in three separate experiments. Data are presented as mean±S.E.M. *P=0.055 *versus* NBSF; **P<0.05 *versus* unstimulated cells; ^#^*P*<0.05 *versus* TGF*β*. (**b**) NBS and HTS fibroblasts were subjected to quantitative real-time PCR (qRT-PCR) analysis of myofibroblast gene expression changes after incubation with or without JQ1 (1 *μ*M) and TGF*β* (10 ng/ml). The experiment was repeated three times. Data are presented as mean±S.E.M. *P<0.05 *versus* NBSF; **P<0.05 *versus* unstimulated cells; ^#^*P*<0.05 *versus* TGF*β*. (**c**) BRD4 knockdown with siRNA was performed in HTS fibroblasts before TGF*β* (10 ng/ml) stimulation for 24 h. Cellular mRNA was extracted and qRT-PCR was performed for BRD4-dependent myofibroblast genes. This experiment was repeated three times. Data are presented as mean±S.E.M. **P<0.05 *versus* control siRNA−TGF*β*; #*P*<0.05 *versus* control siRNA+TGF*β*. (**d**) XChIP analysis for Smad3 and BRD4 on the Nox4 promoter was performed on NBS and HTS fibroblasts incubated with or without TGF*β* (10 ng/ml) for 6 h. Fold change is calculated relative to NBSF−TGF*β* sample. XChIP experiment was performed twice and similar results were observed. Data are presented as mean±S.E.M. (**e**) XChIP for CDK9 binding to the Nox4 promoter. Experiment is carried out as in panel (**d**)

We have previously demonstrated that HTS fibroblasts are hypersensitive to IL-6 trans-signaling and express higher levels of ECM genes and cell proliferation markers,^41^ indicating that HTS fibroblasts are reprogrammed to express the fibrotic phenotype. Therefore, we asked whether HTS fibroblasts also display an exaggerated response to TGF*β* and whether this phenotypic behavior is BRD4 dependent. We observed a greater fraction of myofibroblasts in HTS cultures than NBS under basal conditions (Figure 7a). Importantly, JQ1 treatment reverted most of the myofibroblast population back to quiescent fibroblast state (Figure 7a). Strikingly, incubation with TGF*β* increased the myofibroblast population 4–5-fold in both cell types but co-treatment with JQ1 not only blocked the induction of *α*SMA but also decreased the myofibroblast population to levels observed with JQ1 treatment alone. Nox4, fibronectin and Col1*α*1 expression was significantly elevated (2.5–3-fold) at baseline in HTS fibroblasts *versus* NBS fibroblasts (Figure 7b). Furthermore, TGF*β* induced elevation in mRNA of all four genes but HTS fibroblast displayed an even higher induction (by ~2-fold) of ECM genes fibronectin and Col1*α*1. JQ1 co-treatment blocked TGF*β* induction of myofibroblast genes or even suppressed them below baseline levels. Because we identified SM22*α* and Nox4 gene transcription to be BRD4 dependent in hDFs, we asked whether BRD4 also controlled their transcription in HTS fibroblasts. BRD4 silencing suppressed TGF*β* induction of both SM22*α* and Nox4 by >50% (Figure 7c). Collectively, these data demonstrate that HTS fibroblasts have elevated expression of myofibroblast genes in the presence and absence of TGF*β* and that these cells functionally require BRD4 to maintain their state of transdifferentiation.

### HTS fibroblasts have increased Smad3 and BRD4 binding on the Nox4 promoter

Using XChIP analysis, we observed an ~3-fold increase of Smad3 binding to the Nox4 promoter in HTS cells (Figure 7d), and TGF*β*-induced enrichment of Smad3 was enhanced HTS cells (5.2- *versus* 2.7-fold in NBS fibroblasts). Similarly, there was more constitutive BRD4 binding to the Nox4 gene in HTS fibroblasts – a 2.3-fold increase compared with NBS fibroblasts. TGF*β* also induced enhanced BRD4 on the Nox4 gene in HTS *versus* NBS fibroblasts (5.8- *versus* 2.8-fold, respectively). Similar to the BRD4-binding pattern, there was also more constitutive and TGF*β*-induced CDK9 binding to Nox 4 in HTS fibroblasts *versus* NBS fibroblasts (Figure 7e), indicating hyper-responsiveness of the transcriptional elongation complex in HTS. These data suggest that elevated expression of myofibroblast genes in HTS fibroblasts is due to increased basal accumulation of the Smad3–BRD4 complex promoting enhanced transcription.

## Discussion

HTS is a devastating sequela of burn injury characterized by an overabundance of *α*SMA+ myofibroblasts and excess deposition of ECM. In this study, we verified that persistence of myofibroblasts in HTS dermis is driven by enhanced TGF*β* expression in its microenvironment. Dermal fibroblasts develop *α*SMA+ stress fibers, highly upregulate Nox4 and SM22*α* and increase transcription of fibronectin and Col1*α*1 after exposure to TGF*β*. We demonstrate that Nox4 is involved in *α*SMA+ stress fiber formation and collagen transcription in hDF cells. Nox4 transcription is not only controlled by Smad3 during TGF*β* stimulation but also requires association with the BRD4 epigenetic reader and transcription co-activator. BRD4 interacts with both Smad3 and CDK9, part of P-TEFb complex necessary for phosphorylation on the Ser2 residue of the heptad repeat to induce full-length Nox4 transcripts. We found that HTS fibroblasts express high levels of myofibroblast genes, including Nox4, under basal conditions. HTS have a more robust response of the ECM genes to TGF*β* than NBS fibroblasts – changes that can be inhibited by JQ1. Mechanistically, HTS fibroblasts have more Smad3, BRD4 and CDK9 binding to the Nox4 promoter even in the absence of TGF*β*, suggesting that overactivation of the canonical Smad pathway may be the reason for the hyperresponsive cellular phenotype.

Either inhibition of Nox4 activity with GKT13831 or its suppression with siRNA had limited effect on myofibroblast genes in hDF cells. Nox4 was necessary for induction of *α*SMA and collagen but dispensable for SM22*α* and fibronectin. Previous work using fetal and adult lung fibroblasts^15^ and cardiac fibroblasts^16^ suggested a role of Nox4-generated ROS in the regulation of fibronectin. The discrepancy may reflect phenotypic differences of the fibroblast based on their origin. A global gene expression analysis of human fibroblasts from 43 different anatomical sites suggests systematic differences in the gene expression with respect to anterior–posterior, proximal–distal and dermal–nondermal origins.^22^ Similar to our observations in HTS fibroblasts, in dermal fibroblasts from systemic sclerosis patients, Nox4 was highly expressed and was upstream of Col1 expression.^42^ Surprisingly, Nox4 deficiency in mice delayed wound closure but did not affect the presence of *α*SMA+ myofibroblasts over a 2 week period, suggesting that *α*SMA induction is not entirely Nox4 dependent.^43^

Although TGF*β*-induced Nox4 expression is primarily controlled by a SBE 4 kb upstream of the transcriptional start site of the gene,^44^ our findings that Smad3 inhibition does not completely block Nox4 expression in hDF cells suggests the role of additional regulatory factors. Recent work has identified contributory roles of the CArG box, the binding element of the myocardin-related transcription factor,^45^ as well as the transcription factors TAZ/YAP in the Hippo pathway,^45^ and Sp3^46^ in regulating Nox4 in a cell-type-dependent manner. More work will be required to understand the role, if any, of these transcription factors in hDF cells.

Nevertheless, because Smad3 is an important regulator of Nox4 and the myofibroblast program (Figure 3b), we investigated the role of Smad3 transcription co-activators in this study. We demonstrate for the first time that Smad3 binds to BRD4 in response to TGF*β* stimulation and that BRD4 is functionally required for expression of a subset of Smad3-dependent myofibroblast genes. Although BRD4 is well established to be a component of the PTEFb transcriptional elongation complex,^47, 48^ our studies indicate that BRD4 and CDK9 function in myofibroblast transdifferentiation in independent manners. This conclusion is based on the observations that BRD4 tightly binds to Smad3, whereas CDK9 does not and that BRD4 is dispensable for ECM gene expression, whereas CDK9 is needed. These functional differences are in contrast to the common pattern of BRD4 and CDK9 binding to the Nox4 promoter in HTS and respond to TGF*β* by further enhanced binding to the Nox4 promoter. In addition to its role as an acetylate histone adaptor promoting PTEFb binding to RNA Pol II, BRD4 also facilitates Mediator recruitment to promote transcriptional enhancement and separately is involved as a histone acetyl transferase stimulating nucleosome clearance during transcriptional elongation.^49^ More work will be required to dissect the contributions of these individual activities to myofibroblast gene expression.

The mechanism for Smad3–BRD4 association is not known. BRD4 binds acetylated histones and acetylated transcription factors such as TWIST and RelA/NF-*κ*B, suggesting that acetylated histones and transcription factor serve as important signals for recruitment of transcription machinery important in mesenchymal gene expression programs.^31, 50, 51, 52^ We note that the p300/CBP histone acetyltransferase acetylates Lys-372 and Lys-19 of Smad3 required for Smad3 transcriptional activity. We suspect that one or both of these acetylated residues may serve as docking sites for BRD4, but more work is needed to demonstrate this point. Although JQ1 inhibited all myofibroblast gene expression, BRD4 suppression decreased *α*SMA fibers and Nox4 and SM22 mRNA but not fibronectin and Col1*α*1 mRNA. This finding suggests that other BET proteins such as BRD2 may be involved in myofibroblast transformation of hDFs.^53^

P-TEFb is a complex of CyclinT1 and CDK9 that phosphorylates Pol II on Ser2 to promote transcription elongation during activation of innate immunity.^26^ Here we demonstrate that CDK9 also is important in the expression of myofibroblast genes and specifically for Nox4 expression. TGF*β* induced the enrichment of CDK9 and promoted phosphorylation of Pol II Ser2 on the Nox4 promoter. Although we were unable to detect CDK9 in Smad3 immunoprecipitates, we found that CDK9 suppression inhibited the expression of myofibroblast genes that were also Smad3 dependent. This suggests that Smad3 may not directly interact with CDK9, although CDK9 is functionally required for induction of Smad3-dependent genes.

We have previously demonstrated that HTS fibroblasts express higher levels of gp130 – part of the IL-6 receptor complex – and exhibit an exaggerated response to IL-6 leading to higher expression of Col1*α*2 and fibronectin.^41^ Here we demonstrate that HTS fibroblasts express high levels of Nox4, fibronectin and Col1*α*1 under basal conditions and upregulate the expression even more when exposed to TGF*β*. Our data suggest that increased basal activation of the Smad pathway coupled with BRD4 on the Nox4 gene promoter drives the myofibroblast phenotype in HTS cells. Recently, it has been reported that JQ1 administration before or after onset of hepatic fibrosis in a mouse model limits ECM deposition and decreases myofibroblast markers.^54^ Collectively, these finding support an important role of Smad signaling and BET proteins, including BRD4, in myofibroblast transdifferentiation and fibrosis. These findings suggest that inhibitors of the Smad3–BRD4 pathway may be useful in limiting HTS after burn injury.

## Materials and methods

### Cell culture and tissue biopsies

Biopsies of non-burned skin and postburn HTSs were acquired from pediatric burn patients (aged 0–18 years) at 12, 24 and 48 months following burn injuries covering at least 30% of the total body surface as part of a study approved by IRB at the University of Texas Medical Branch. After obtaining patient consent, biopsies were taken from the site of HTS or the adjacent NBS region during surgical revision procedure. Samples were formalin fixed, paraffin embedded and sectioned at 4 *μ* thickness. Fibroblasts from HTS and NBS biopsies were isolated as previously described^55^ and propagated in DMEM containing 15% fetal bovine serum (FBS) and 1% antibiotic/antimycotic (Invitrogen, Carlsbad, CA, USA). Experiments were performed on NBS and HTS cells from equivalent 6–15 passages. Cells were not tested for mycoplasma contamination. For experiments involving TGF*β*, NBS and HTS cells were serum starved overnight in media containing 0.5% FBS. Normal adult hDF were purchased from Lonza and cultured in the manufacturer recommended media. All experiments with hDF cells were performed on cells from passage 4 to 12.

### Reagents and antibodies

Human TGF*β*1 (Peprotech, no. 100-21) was suspended in vehicle containing BSA, as recommended by the manufacturer, at 10 *μ*g/ml stock concentration. Aliquots were kept frozen at −20 °C until used. GKT137831 was a gift from Genkyotex (Geneva, Switzerland), JQ1 and LY2157299 were purchased from Apexbio (Houston, TX, USA) and Can508 was purchased from Santa Cruz (Dallas, TX, USA).

The following primary antibodies were used for immunohistochemistry (IHC) or immunofluorescence: anti-TGF*β* (Abcam, ab66043, 1:100), anti-SMA (Abcam, Cambridge, MA, USA, ab5694, 1:100), anti-Smad2/3 (Cell Signaling, no. 8685, 1:300), anti-phospho-Smad2/3 (Cell Signaling, Danvers, MA, USA,no. 8828, 1:200), anti-BRD4 (Millipore, Tenecula, CA, USA, ABE1391, 1:100), and anti-CDK9 (Santa Cruz, sc-484, 1:100). Fluorescent secondary antibodies utilized were highly cross-adsorbed goat anti-rabbit IgG or goat anti-mouse IgG conjugated to AlexaFluor (AF)-568 or AF-488 (Molecular Probes, Eugene, OR). Cell Signaling antibodies (Anti-Smad2/3 and anti-phospho-Smad2/3) and Santa Cruz antibody (anti-CDK9) were also used for western blottings. Other antibodies used in western blotting analyses were raised against BRD4 (Invitrogen, 23476, 1:1000), GAPDH (Millipore, MAB374, 1:1000) and *β*-actin (Sigma St. Louis, MO, USA, A5316, 1 : 5000) or were HRP-conjugated secondary antibodies (GE Healthcare, Pittsburgh, PA, USA) raised against rabbit or mouse IgGs. For XChIP analysis, anti-P-Smad3 (Cell Signaling, no. 9520), anti-BRD4 (Millipore), anti-CDK9 (Santa Cruz) and anti-Phospho-Pol II (Abcam, ab5095) antibodies were used.

### Quantitative RT-PCR

hDF and NBS/HTS fibroblasts were seeded in six-well plates at 80% confluency and serum starved overnight with media containing 1% FBS. For experiments involving inhibitors, LY2157299 (10 *μ*M), GKT137831 (20 *μ*M) and JQ1 (0.5-1 *μ*M) were added to media 1 h before TGF*β* while Can508 (30 *μ*M) was added 6 h before to ensure maximum inhibition.^24^ Fibroblasts were stimulated with hTGF*β*1 for 24–48 h. Cellular RNA was extracted from fibroblasts using Tri Reagent (Sigma) according to the manufacturer's instructions and 1 *μ*g RNA was reverse transcribed using Super Script III First-Strand Synthesis System (Invitrogen). The resultant cDNA was diluted 1 : 2 with RNAase free water and 1 *μ*l cDNA was amplified in a 10 *μ*l reaction containing 5 *μ*l SYBR Green Super Mix and 500 nM primers. The reaction mixtures were aliquoted in triplicates into a Bio-Rad 96-well plate. Bio-Rad CFX96 Real-Time thermal cycler was used to run the real-time reactions according to the following protocol: 95 °C for 3 min, followed by 40 cycles of 10 s at 95 °C, 30 s at 55 °C, and the reaction was terminated at 95 °C for 10 s. PCR products were subjected to melting curve analysis to ensure that only a single product was formed. Changes in gene expression were determined using ΔΔCT method. DNA Polymerase *β* was utilized as the housekeeping gene. The human primers used in the qPCR reactions were SM22 forward: 5′-CCGTGGAGATCCCAACTGG-3′, SM22 reverse: 5′-CCATCTGAAGGCCAATGACAT-3′ Nox4 forward: 5′-CAGATGTTGGGGCTAGGATTG-3′, Nox4 reverse: 5′-GAGTGTTCGGCACATGGGTA-3′ fibronectin forward: 5′-AGGAAGCCGAGGTTTTAACTG-3′, fibronectin reverse: 5′-AGGACGCTCATAAGTGTCACC-3′ Col1*α*1 forward: 5′-GTGCGATGACGTGATCTGTGA-3′, Col1*α*1 reverse: 5′-CGGTGGTTTCTTGGTCGGT-3′ Smad3 forward: 5′-CCATCTCCTACTACGAGCTGAA-3′, Smad3 reverse: 5′-CACTGCTGCATTCCTGTTGAC-3′ BRD4 forward: 5′-ACCTCCAACCCTAACAAGCC-3′, BRD4 reverse: 5′-TTTCCATAGTGTCTTGAGCACC-3′ CDK9 forward: 5′-GGGCTGTTGAGCAATGTTTTG-3′, CDK9 reverse: 5′-GCAGGATCTTGTTTCTGTGGA-3′ and DNA polymerase *β* forward: 5′-CCGCAGGAGACTCTCAACG-3′, DNA polymerase *β* reverse: 5′-GTACTTGTGGATAGCTTGGCTC-3′.

### Western blotting

Fibroblast cells were scraped into Eppendorf tubes and lysed in RIPA buffer (150 mM NaCl, 1% Triton X-100, 0.5% sodium deoxycholate, 0.1% SDS, 50 mM Tris, pH 8) containing protease (Sigma, P8340) and phosphatase inhibitor cocktails (Thermo Scientific, San Jose, CA, USA). Cellular extracts were sonicated for 10 s to ensure complete cell lysis and kept on ice for 30 min. DNA was pelleted by centrifugation at 12 000 × *g* at 4 °C for 20 min, and the supernatant was transferred to a separate tube. Protein samples were kept frozen at −80 °C until needed for analysis.

Protein concentrations were measured using Bradford Protein assay (Protein Reagent, Bio-Rad, Hercules, CA, USA). In all, 30–50 *μ*g protein was fractionated by 10% SDS-PAGE and transferred to PVDF membrane. After blocking with 5% milk in TBS-Tween (TBS-T) for 1 h, membranes were incubated with primary antibody overnight at 4 °C. Membranes were washed thoroughly the next day in TBS-T and incubated with HRP-conjugated secondary antibody for 1 h. ECL Western blot solution (Amersham, Piscataway, NJ, USA) was used as the chemiluminescence substrate and the exposed X-ray film was developed with a Kodak machine (Rochester, NY, USA).

### IHC on tissue sections

Tissue sections of skin biopsies from HTS and NBS regions were deparaffinized and rehydrated and antigen retrieval was performed with 10 mM sodium citrate, pH 6. Sections were blocked with 5% goat serum in TBS-T for 1 h and incubated with primary antibody overnight at 4 °C. After washing three times with TBS-T, sections were incubated with a fluorescent secondary antibody (AF-568 goat anti-rabbit) for detection of αSMA or a biotinylated secondary antibody for detecting TGF*β* for 1 h. For detection via immunofluorescence, tissue sections were washed again with TBS-T, incubated with DAPI (Molecular Probes) for nuclear counterstaining and glass coverslips were mounted using Dako Fluorescence Mounting Medium (Carpenteria, CA, USA). For chromogen-based detection, tissue sections were incubated with avidin–biotin complex (Vector Labs, PK6101) followed by exposure to DAB substrate (Vector Labs, Burlingame, CA, USA, SK4100) and counterstaining with hematoxylin. Sections were dehydrated with serial washes in ethanol and xylene before mounting of coverslips. Immunostained sections were evaluated using a Nikon Eclipse 80i microscope (Melville, NY, USA) and images were captured with an attached Nikon DSM1200F digital camera. ImageJ (NIH, Bethesda, MD, USA) software was used to make composite images and to add the scale bar.

### Immunocytochemistry

Fibroblast cells were seeded on coverslips at 30–40% confluency, serum starved for 16 h and stimulated with TGF*β* (10 ng/ml) in the presence or absence of an inhibitor. Similar to the qRT-PCR experiments, cells were preincubated with LY2157299 (10 *μ*M), GKT137831 (20 *μ*M), JQ1 (0.5-1 *μ*M) or Can508 (30 *μ*M) before TGF*β* stimulation. After 24–48 h incubation, cells were fixed in 4% paraformaldehyde and permeabilized with 0.25% Triton X-100. Cells were then blocked with 5% goat serum for 1 h and incubated with primary antibody overnight at 4 °C, followed by 1 h incubation with AF-568 or AF-488 conjugated secondary antibody. For detection of filamentous actin, AF-568-conjugated phalloidin (Molecular Probes) was added to cells after secondary antibody treatment. Cells were counterstained with DAPI and mounted on to glass slides for analysis. ImageJ (NIH) software was used to make composite images and to add the scale bar.

### ROS detection assay

Nox4 activity and ROS were assessed using a DCF-DA assay as described previously with some modifications.^29^ Briefly, control siRNA or Nox4 siRNA-treated cells were seeded at a density of 35 K cells/well in a 24-well plate and serum starved before being treated with TGF*β*. Cells were washed 2 × with PBS and incubated with 10 *μ*M DCF-DA (Molecular Probes) for 30 min in phenol-red-free media. Following incubation with the dye, cells were washed again 2 × with PBS and incubated for another hour with fresh media. Plates were read using a Tekan Infinite F200 Pro fluorescence plate reader (ex: 480 nm, em: 520 nm). Each treatment was assessed in quadruplicate and background reading was subtracted to attain the cellular DCF fluorescence value. hDF cells treated with hydrogen peroxide (4.8 nM) for 1 h were used as positive control.

### Collagen gel contraction assay

Collagen gel contraction assay was performed as previously reported.^56^ Briefly, fibroblasts were harvested from 10-cm plates and re-suspended in DMEM containing 1% FBS. Fifty thousand fibroblasts were then seeded into collagen matrices along with GKT137831 (20 *μ*M), JQ1 (1 *μ*M) or Can508 (30 *μ*M) and cast into wells of a 24-well plate. The collagen gels were released from the edges and left floating in DMEM+1% FBS±inhibitors. Experimental groups were assessed in triplicate. Gels were photographed at 0, 24 and 48 h. Calculations based on the 48 h time point are reported in this manuscript. ImageJ software (NIH) was used to calculate the change in surface area, which is reported as the percentge of gel contraction.

### Co-immunoprecipitation

Fibroblasts cells were treated with TGF*β* (10 ng/ml) for 24 h before being lysed with RIPA buffer containing protease inhibitor cocktail (Sigma). Equal amounts of protein were incubated with 4 *μ*g of control IgG or an antigen-specific antibody overnight at 4 °C under constant agitation. Next day, prewashed protein-A-conjugated magnetic beads (Invitrogen) were added to each sample and samples were incubated for 2 h at 4 °C on a tube rotator. Magnetic beads were washed 3 × with RIPA buffer and once with PBS. Immune complexes attached to the magnetic beads were pelleted and kept frozen at −80 °C until used for analysis with SID-SRM-MS.

### Stable isotope dilution-selected reaction monitoring-mass spectrometry

SID-SRM-MS assays of SMAD3, CDK9 and BRD4 were developed using a workflow described in previous publications.^34, 35, 36^ The signature peptides and SRM parameters are listed in Table 1. The peptides were chemically synthesized incorporating isotopically labeled [^13^C_6_^15^N_4_] Arginine or [^13^C_6_^15^N_2_] Lysine to a 99% isotopic enrichment (Thermo Scientific). The proteins immunoprecipitated with anti-SMAD3 and anti-BRD4 antibodies were captured by protein A magnetic beads (Dynal Inc., Carlsbad, CA, USA). The proteins on the beads were digested with trypsin as described previously.^34, 35, 36^ Briefly, beads were washed with PBS 3 × and then resuspended in 100 *μ*l of 50 mM ammonium hydrogen carbonate (pH 7.8) and 40 *μ*l of 0.1 *μ*g/*μ*l of trypsin was added. The samples were mixed and trypsinized by gentle vortexing overnight at 37 °C. After digestion, the supernatant was collected. The beads were washed with 50 *μ*l of 50% acetonitrile (ACN) three times and the supernatant was pooled and dried. The tryptic digests were then reconstituted in 50 *μ*l of 4% ACN-0.01% TFA. An aliquot of 5 *μ*l of diluted stable isotope-labeled signature peptides was added to each tryptic digest. These samples were desalted with ZipTip C18. The peptides were eluted with 80% ACN and dried with Speedvac. The peptides were reconstituted in 30 *μ*l of 5% formic acid–0.01% TFA and were directly used for LC-SRM-MS analysis without further purification or fractionation. SRM assays were performed with a TSQ Vantage triple quadrupole mass spectrometer equipped with nanospray source (Thermo Scientific) as described previously.^34, 35, 36^ The online chromatography were performed using an Eksigent NanoLC-2D HPLC system (AB SCIEX, Dublin, CA, USA). An aliquot of 10 *μ*l of each of the tryptic digests was injected on a C18 reverse-phase nano-HPLC column (PicoFrit, 75 *μ*m × 10 cm; tip ID 15 *μ*m) at a flow rate of 500 nl/min with a 20-min 98% A, followed by a 15-min linear gradient from 2 to 30% mobile phase B (0.1% formic acid–90% ACN) in mobile phase A (0.1% formic acid). The TSQ Vantage was operated in high-resolution SRM mode with Q1 and Q3 set to 0.2 and 0.7-Da Full Width Half Maximum. All acquisition methods used the following parameters: 2100 V ion spray voltage, a 275 °C ion transferring tube temperature, a collision-activated dissociation pressure at 1.5 mTorr, and the S-lens voltage used the values in S-lens table generated during MS calibration.

All SRM data were manually inspected to ensure peak detection and accurate integration. The chromatographic retention time and the relative product ion intensities of the analyte peptides were compared with those of the stable isotope-labeled standard (SIS) peptides. The variation of the retention time between the analyte peptides and their SIS counterparts should be within 0.05 min, and the difference in the relative product ion intensities of the analyte peptides and SIS peptides were <20%. The peak areas in the extract ion chromatography of the native and SIS version of each signature peptide were integrated using Xcalibur 2.1 (Sugarland, TX, USA). The default values for noise percentage and baseline subtraction window were used. The ratio between the peak area of native and SIS version of each peptide was calculated.

### siRNA knockdown of mediators of myofibroblast transdifferentiation

hDF or HTS fibroblast were grown to 90% confluency before being harvested with 0.25% Trypsin. After trypsin neutralization, 1 × 10^6^ cells were electroporated with 100 pmol of non-specific control siRNA (ON-TARGET plus SMARTpool by Dharmacon) or gene-specific siRNA (ON-TARGET plus SMARTpool by Dharmacon (Lafayette, CO, USA) targeting Nox4, Smad3, BRD4 or CDK9) using U-023 program (Amaxa, Walkersville, MD, USA). Cells were seeded in six-well plates or on coverslips. After 48 h, cells were serum starved overnight and then treated with TGF*β* (10 ng/ml) for 24–48 h. Target knockdown by at least 75% by qRT-PCR was needed for experimental inclusion.

### Dual crosslink chromatin immunoprecipitation

XChIP was performed as previously described.^57^ Briefly, fibroblast cells (~1.2 × 10^6^ cells per 10-cm plate) were washed 2 × with PBS; protein–protein crosslinks were made with 2 mM disuccinimidyl glutarate (Pierce, Carlsbad, CA, USA); and protein–DNA crosslinks were formed with formaldehyde. Chromatin was sheared via five rounds of sonication at setting 4 with 10 s breaks on ice between pulses (Branson Sonifier 150, Branson Ultrasonics, Danbury, CT, USA). Equal amount of sheared chromatin was immunoprecipitated overnight at 4 °C with 4 *μ*g of control IgG or target-specific antibody (anti-Smad3, -P-Smad3, -BRD4, -CDK9 or -P-Pol II). Protein A-conjugated magnetic beads (Invitrogen) were added to capture antibody–antigen complexes. Immunoprecipitates were washed and then eluted with elution buffer (0.09 M NaHCO_3_, 1% SDS). Samples were de-crosslinked in 0.2 M NaCl at 65 °C for 2–3 h. DNA was isolated using phenol–chloroform extraction and ethanol precipitation and re-suspended in TE buffer. Real-time genomic PCR was performed on the isolated DNA using primers specific for the Nox4 promoter: Nox4 forward 5′-GGACATCCTGAACAGCAGCA-3′ and Nox4 reverse 5′-CTGCACCAGTCTGCTCCG-3′. Fold change of DNA in each sample was determined by first normalizing the absolute value to the input DNA reference and then calculating the fold change relative to unstimulated cells.

### Statistical analysis

Experiments were repeated at least three times, and the mean±S.E.M. of *n*=3 experiments was plotted, unless otherwise noted. This sample size was selected based on effect sizes and experimental variability. Differences across multiple groups were analyzed by analysis of variance, followed by Newman–Keuls or Tukey's pairwise comparison. SigmaPlot 12.5 (Systat Software Inc., San Jose, CA, USA) was used for analyzing and graphing data. *P*<0.05 was considered statistically significant.

## Figures and Tables

**Table 1:** Signature peptides and SRM transitions for SRM analysis of SMAD3 and BRD4

**Protein**	**SRM peptide sequence**	**Precursor** ***m*****/*****z***	**Product** ***m*****/*****z***	**CE (V)**	**Precursor** ***Z***	**Product** ***Z***	**Ion type**
SMAD3	VETPVLPPVLVPR	708.434	777.497	27	2	1	y7
			890.582	27	2	1	y8
			989.650	27	2	1	y9
			1086.70	27	2	1	y10
	FC[Carboxyamidomethyl]LGLLSNVNR	646.842	815.473	25	2	1	y7
			702.389	25	2	1	y6
			872.494	25	2	1	y8
			985.578	25	2	1	y9
	GLPHVIYC[Carboxyamidomethyl]R	557.795	710.365	22	2	1	y5
			847.424	22	2	1	y6
			944.477	22	2	1	y7
			1057.561	22	2	1	y8
BRD4	AASVVQPQPLVVVK	717.938	1106.693	28	2	1	y10
			782.513	28	2	1	y7
			879.566	28	2	1	y8
			1007.624	28	2	1	y9
	DAQEFGADVR	554.257	664.341	22	2	1	y6
			793.383	22	2	1	y7
			921.442	22	2	1	y8

Abbreviations: *m*/*z*, mass-to-charge ratio; *Z*, charge state
